# Supplementary material for: A contrastive adversarial encoder for multi-omics data integration
Source: PLoS One. 2025 Oct 16;20(10):e0333134. doi: 10.1371/journal.pone.0333134 (PMC12530548; doi:10.1371/journal.pone.0333134)
Supplement: Attachment — Submitted filename: Response to Reviewers (PONE).docx [file pone.0333134.s001.docx]

**Ref: Submission ID PONE-D-25-24854**

Decision: Revision

Article Title: “A Contrastive Adversarial Encoder for Multi-Omics Data Integration”

To: PLOS ONE Editor

Re: Response to reviewers

**Dear Editor,**

Thank you for allowing revision and resubmission of our manuscript, with an opportunity to address the reviewers’ comments.

We are uploading (a) our point-by-point response to the comments (below) (response to reviewers, under “Author’s Response Files”), (b) an updated manuscript with yellow highlighting indicating changes (“Revised Manuscript with Track Changes.pdf ”), and (c) a clean updated manuscript without highlights (“Manuscript.pdf”).

Best regards,

<Yang Heng> et al.

**Reviewer 1# Concern # 1 (Results are based on a single train-test split without variance estimates or statistical tests. This undermines the reliability of performance comparisons.):**

**Author response:** Thank you very much for your insightful comment. We fully agree that relying on a single train-test split is insufficient for demonstrating robust performance. In fact, all our experiments were conducted using 5-fold cross-validation with different random seeds to ensure statistical reliability. However, we acknowledge that this was not clearly stated in the 3.2“Training and Hyper-parameters Setting” section, and we have now revised the text to clarify our experimental setup.

Additionally, we appreciate your observation regarding the missing variance estimates. In Figure 3, due to space constraints, we initially omitted the standard deviation values. We have now updated the figure format and tables to explicitly include the mean ± standard deviation for all reported metrics.

**Reviewer#1, Concern # 2 (Despite a complex model, there is no validation loss reporting or regularization discussion. Overfitting cannot be ruled out.):**

**Author response:** Thank you for highlighting this important point. We agree that reporting validation loss or regularization strategies is essential to assess overfitting risk. Although the original manuscript did not explicitly present validation loss curves, we did use a validation set during training and monitored validation performance in each epoch. In addition, we applied L2 regularization (weight decay) and gradient clipping to mitigate overfitting and stabilize training, as now explicitly stated in the revised 3.2 “Training and Hyper-parameters Setting” section. These further ensure the robustness of our model and prevent overfitting despite its complexity.

**Reviewer#1, Concern #3 (Claims about handling imbalance and modality synergy are not supported by class-wise metrics or ablation studies.):**

**Author response:** We greatly appreciate the reviewer’s valuable feedback. Regarding the model's ability to handle data imbalance, our ablation study in Section 3.4 provides strong evidence. The incorporation of the contrastive learning (CL) module significantly improves overall performance (Acc/F1 +9%), particularly by addressing data imbalance through an implicit balancing mechanism. Specifically, CL forces the model to focus on the similarity between samples rather than simply relying on class frequency. This is achieved by constructing positive and negative sample pairs, which ensures that the model learns more robust features regardless of class distribution.

Further indirect evidence from our ablation study supports this. When CL is removed (CAEncoder_notCL), the model’s performance drops significantly (F1 ↓ 0.085), and the prediction variance increases (±0.0389 Acc). This indicates that CL constrains the model's overfitting to the majority class, indirectly helping to preserve the generalization capability for minority classes. This demonstrates that the CL module not only improves overall performance but also mitigates the detrimental effects of class imbalance.

**Reviewer#1, Concern 4 (Competing methods are not re-evaluated under the same setup, limiting fairness of comparison. ):**

**Author response:** Thank you for your valuable feedback. We fully understand that fair comparison requires all competing methods to be evaluated under the same experimental setup. However, due to practical constraints such as the unavailability of source code, incomplete descriptions of preprocessing procedures, or restricted access to specific model implementations, we were unable to re-implement all baseline methods under an identical environment. Therefore, for these methods, we reported the results as published in their original papers using the same dataset and evaluation metrics. In future work, we plan to re-implement more baseline methods under a unified setup and conduct more comprehensive and fair comparisons.

**Reviewer#2, Concern #1 (This study is an application of machine learning in cancer prediction. However, the article didn’t reveal any biological/medical updates. What did we learn about those cancers from the current study?):**

**Author response:** Thank you for pointing out the insufficient discussion on biomedical insights in our study. We fully understand that medical research not only requires methodological advancements but also a deeper understanding of disease mechanisms.

Although the core contribution of this work lies in proposing a novel multi-omics data fusion framework for cancer classification and prediction, we believe our study provides meaningful implications for cancer research in the following aspects:

Data Integration Perspective:

Our proposed model effectively integrates data from multiple omics layers (e.g., mRNA expression, CNV, protein levels) and constructs a collaborative latent representation space. This enhances the comprehensive understanding of tumor phenotypes and offers a reliable data-driven foundation for subsequent mechanistic studies.

Modal Balance and Collaborative Modeling:

By leveraging contrastive learning and the CycleGAN mechanism, our method mitigates the issue of modality dominance during data fusion. It ensures that weaker modalities are also effectively utilized, which is critical for uncovering potential biological signals hidden in non-dominant omics layers.

Improved Precision in Classification:

The proposed approach achieves significantly higher classification accuracy than existing models across multiple cancer datasets. This enhanced discriminative ability can contribute to better identification of cancer subtypes, thereby supporting personalized treatment decisions and prognosis evaluation.

**Reviewer#2, Concern #2 (This study is not complete without discussing how the trained model learned discriminative features and improved generalization. Listing high accuracy and F1 score is not enough. Such proof should be in a data driven approach.):**

**Author response:** Thank you for pointing this out. To address this, we conducted detailed ablation experiments to evaluate the individual contributions of each core component in our proposed model (see Table 5). By removing the contrastive loss and CycleGAN components respectively, we observed significant performance drops, which clearly demonstrate that each module plays a critical role in enhancing both the discriminative capability and generalization performance of the model.

- The contrastive loss significantly improves intra-class compactness and inter-class separability in the feature space.

- The CycleGAN enables effective domain-level feedback and preserves modality-specific structure during transformation, reducing redundancy and promoting generalization.

These results indicate that our model does not rely on superficial performance gains but indeed learns meaningful and robust representations in a data-driven.

**Reviewer#2, Concern # 3 (It’s not clear how the CycleGAN was coupled with the ViT during training and inference. The model structure diagram is very confusing. More details are needed regarding the end-to-end design.):**

**Author response:** Thank you for your insightful comment. We appreciate the opportunity to clarify the integration between the Vision Transformer (ViT) encoder and the CycleGAN modules. Our model is trained in an end-to-end fashion, where both ViT and CycleGAN are jointly optimized through a unified loss function that includes contrastive loss, adversarial loss, and cycle-consistency loss. During inference, only the ViT encoder is used for downstream classification tasks. These clarifications have been added to the revised manuscript. A detailed explanation has been added at Section 2 (“Proposed Model”)、Section 2.1 (“ViT Encoder”)、Section 2.2 (“CycleGAN Architecture”).

**Reviewer#2, Concern # 4 (It’s not clear how CycleGAN provides feedback to the ViT encoder. More details are needed. ):**

**Author response:** Thank you for pointing this out. We have clarified that the feedback from CycleGAN is realized through the backward propagation of adversarial and cycle-consistency losses. Since the generator 𝐺1 takes the encoder output 𝑧=(𝑥) as input, and the generator 𝐺2 attempts to reconstruct 𝑧 from the synthetic data, the losses from both generators and discriminators are functions of the encoder output. Therefore, these losses provide indirect but effective gradient feedback to the ViT encoder during training, enhancing its representation learning.

**Author action:** We have Added a paragraph at the end of Section 2.1 (“ViT Encoder”) to explain how the CycleGAN losses are backpropagated to the encoder. Supplemented the explanation at the end of Section 2.2 (“CycleGAN Architecture”) to explicitly highlight how the encoder benefits from feedback through CycleGAN’s loss functions. Also reiterated this mechanism in the closing of Section 2.3 (“Loss Functions”) to emphasize the feedback loop and its effect on encoder optimization.

**Reviewer#2, Concern # 5 (It’s not clear if one universal model was developed or separate model was trained on each dataset. ):**

**Author response:** Thank you for your important question. We confirm that a universal model was developed and trained jointly across all datasets, rather than training separate models for each dataset.

**Reviewer#2, Concern # 6 (The affect of estimator number on Random forest model performance doesn’t make sense. If designed properly, the model performance won’t drop with further increasing number of estimators due to the nature of random selection of sample and features. ):**

**Author response:** Thank you for pointing out this important issue. In our initial experiments, we observed a decrease in model performance as the number of estimators increased. This phenomenon could be attributed to the small sample size and inherent randomness during model training.

To further validate this, we conducted more stringent controls in our experiments and tested on different datasets. The new results show that, as the number of estimators increases, the model's performance remains generally stable, with only slight fluctuations, which aligns with the theoretical behavior of random forests.

We have revised the corresponding section of the manuscript based on these findings to more accurately reflect the model's performance.

**Reviewer#2, Concern # 7 (The presented information is far less than enough to reconstruct the model. Most of key information for model architecture are missing, like depth and size of MLP. ):**

**Author response:** Thank you for your insightful feedback. We fully recognize the importance of providing sufficient architectural details to ensure reproducibility and clarity. In response, we have added key implementation details at the end of Section 2.1 (ViT Encoder), including the number of Transformer blocks, attention heads, embedding dimensions, and the layer-wise structure of the MLP. These additions aim to facilitate a more accurate reconstruction and understanding of the proposed model.

**Reviewer#2, Concern # 8 (Were model structures and hyper parameters fine tuned? Model structures weren’t fully disclosed so it’s unknown if and how the model structures were decided. Hyper parameters seem not tuned according to the limited information about hyper parameters, like n_epoch=100. ):**

**Author response:** Thank you for your valuable feedback.

Hyperparameter tuning strategy: Although our primary goal was to ensure consistent evaluation across different datasets, we performed a limited grid search on a subset of the training data. We experimented with various learning rates (e.g., 1e-3, 1e-4, 5e-5), batch sizes (32,64,128), and numbers of epochs (80-150 ), and selected the combination that yielded the best validation performance (learning rate = 1e-4, batch size = 64, epochs = 100).

Statistical robustness control: To ensure stable and generalizable results, we adopted 5-fold cross-validation and repeated the training process with five different random seeds. Additionally, gradient clipping and L2 regularization were applied to prevent overfitting and stabilize the training process.

**Reviewer#2, Concern # 9 (All the data cited in this study should be proofread. For example, the results cited from reference 37 don’t match with the original publication at all.):**

**Author response:** Thank you very much for your valuable feedback. We fully acknowledge the importance of rigor and accuracy in citing previous work. Upon careful re-examination, we identified the source of the discrepancy was due to a miscommunication among team members during data compilation, which unfortunately led to an erroneous reporting of results from Reference 37. We have thoroughly reviewed and corrected the relevant data in the manuscript. Additionally, we have conducted internal discussions to strengthen our data verification and review procedures to prevent similar issues in the future. We sincerely apologize for this oversight and appreciate the reviewer’s careful scrutiny that helped us improve the quality of our work.

**Reviewer#2, Concern # 10 (The authors need to address why it is reasonable to mix modalities from patients with same cancer type. Doing so creates a biologically inconsistent sample. ):**

**Author response:** Thank you for your valuable feedback. To further justify the feasibility of this approach, we provide an explanation based on our ablation study results. First, our ablation study (Table 5) has demonstrated that incorporating contrastive learning significantly improves the classification performance of the model. Since contrastive learning requires inputs consisting of an anchor, a positive sample, and a negative sample, the model must adopt a specific sample construction strategy. If our method of constructing positive and negative samples were unreasonable, the model’s performance would not improve with contrastive learning and might even be adversely affected. However, the experimental results clearly show that after integrating contrastive learning, the model becomes more effective at distinguishing different cancer subtypes. This indicates that our sample construction strategy is reasonable and plays a crucial role in enhancing the model’s generalization capability.

To further address the issue of biological consistency, it is important to note that while there is inter-patient heterogeneity, omics profiles from patients with the same cancer type often share common core molecular features and pathway-level characteristics. Therefore, treating multi-omics data from different patients with the same cancer as positive pairs is a biologically reasonable approximation, especially when aiming to model subtype-specific patterns rather than patient-specific noise.

Moreover, our objective is to learn modality-invariant, cancer-type-discriminative representations rather than individual patient signatures. The significant performance improvement observed in our ablation study further confirms that this strategy does not introduce harmful noise, but rather facilitates generalizable representation learning.

**Reviewer#3, Concern # 1 (The authors used a ViT encoder to integrate multi-omics data. Does this include genomic, transcriptomic, proteomic, and metabolomic data? Genomic data are often binary, while others are continuous—how were these heterogeneous data types handled during model training?):**

**Author response:** Thank you to the reviewer for this valuable question. Our study indeed employs multi-omics data for integrative analysis, covering different layers of omics information, but it does not include metabolomics data. Specifically:

In Dataset-1, we utilized copy number variation (CNV), mRNA expression, and proteomics (RPPA) data, representing genomic, transcriptomic, and proteomic information, respectively. In *Dataset-2* and *Dataset-3*, we incorporated DNA methylation, miRNA expression, and single nucleotide variation (SNV) data, covering a wide range of molecular modalities from epigenetic regulation to genomic mutations. However, metabolomics data were not included in this study. In future work, we plan to incorporate metabolomics data to further enhance the generalizability of the proposed model.

To address the heterogeneity in data types across omics modalities—such as the discrete/binary nature of CNV or SNV data versus the continuous nature of mRNA, protein, and miRNA expression—we performed a unified preprocessing pipeline prior to model training. This included converting all categorical variables (e.g., CNV, SNV) into numerical formats and applying standardization (zero mean and unit variance) to all features. These steps were designed to reduce the scale discrepancies among different omics types and ensure comparability in numerical range across modalities. Additionally, when constructing positive and negative sample pairs, we accounted for the sample distribution across different cancer types and omics modalities, thus building a balanced set of training pairs to mitigate the adverse effects of sample imbalance.

In summary, through a unified and standardized data preprocessing framework, we effectively alleviated the heterogeneity in data types, scales, and sample distributions across multi-omics datasets, thereby enhancing the model’s adaptability and robustness in integrative analysis.

**Reviewer#3, Concern # 2 (Except for genomics, most omics data types (e.g., transcriptomics, proteomics) are highly susceptible to batch effects across batches or populations. Could the authors introduce how they addressed batch effects in the preprocessing or modeling pipeline?):**

**Author response:** Thank you to the reviewer for highlighting the issue of batch effects, which is indeed a critical and well-recognized challenge in multi-omics analysis. In this study, we did not employ traditional batch correction methods explicitly, as the datasets we used (e.g., TCGA and ROSMAP) had already undergone basic normalization procedures. Nonetheless, we took the following measures during preprocessing to further mitigate the potential impact of batch effects:

First, we applied Z-score standardization (zero mean and unit variance) separately within each omics modality (e.g., mRNA, RPPA, miRNA) to remove systematic biases caused by differences in measurement scales across platforms or modalities.

Second, the design of the contrastive learning framework itself provides a degree of robustness to batch effects. During training, anchor, positive, and negative samples are constructed across patients and across modalities. In particular, positive pairs are often composed of data from different individuals but belonging to the same cancer subtype, while negative pairs contain mixed-modality data from different classes. This pairing strategy inherently increases the model’s tolerance to non-biological variations such as batch effects and encourages it to focus on meaningful biological signals.

Finally, the mechanism of sample pair construction introduces training perturbations that help reduce systematic biases. For instance, by constructing positive pairs using different patients’ modalities, we introduce a form of “heterogeneity” into the data. As a result, even in the presence of minor batch effects, the model is compelled to learn discriminative and essential biological features rather than relying on modality-specific numerical patterns.

In summary, although we did not explicitly apply batch effect correction algorithms, our model benefits from intra-modality normalization, the robustness of the contrastive learning framework, and perturbation-based sample construction strategies. These collectively enhance the model’s resilience to batch-related noise. In future work, we plan to further investigate the integration of batch correction methods within multi-omics contrastive learning frameworks to improve both model stability and interpretability.

**Reviewer#3, Concern # 3 (Following from the previous point, normalization is a critical step in omics data analysis. How did the authors normalize different omics layers prior to integration, and was this done separately or jointly?):**

**Author response:** In our study, we performed standardization independently within each omics modality (e.g., mRNA, RPPA, miRNA), rather than combining all modalities for joint processing. This per-modality Z-score normalization was applied to mitigate differences in scale and distribution arising from distinct measurement platforms and technologies. This strategy ensures that each modality contributes comparably during model training, while preserving biologically meaningful variation unique to each omics layer. In future work, we plan to explore joint normalization strategies for scenarios requiring tighter cross-modality alignment.

**Reviewer#3, Concern # 4 (The authors chose CycleGAN instead of other GAN architectures. Could they provide a brief introduction for this choice and explain what advantages CycleGAN offers in the context of multi-omics integration?):**

**Author response:** Thank you for your valuable feedback. We chose CycleGAN over other GAN architectures because it is capable of learning bidirectional mappings under unpaired data conditions and effectively preserves structural consistency between different omics modalities through the use of cycle consistency loss. In contrast, traditional GANs typically rely on strictly paired data for training, which is often impractical in multi-omics data integration scenarios and poses significant limitations. Therefore, CycleGAN offers a more flexible and effective solution for our study.

**Reviewer#3, Concern # 5 (Deep learning models typically require large sample sizes, yet multi-omics studies often suffer from limited sample availability. Could the authors give a short discussion about how sample size impacted model training and whether any strategies (e.g., data augmentation, regularization) were used to mitigate overfitting?):**

**Author response:** Thank you for your valuable comment. We agree that limited sample size is a common challenge in multi-omics studies, which may increase the risk of overfitting in deep learning models. In this study, we mainly addressed this issue through the use of L2 regularization. This strategy effectively prevents the model from memorizing noise in the training data, thereby improving its generalization ability to unseen samples. Furthermore, the contrastive learning mechanism we introduced encourages the model to learn discriminative features between samples rather than relying heavily on large amounts of labeled data, which also helps mitigate overfitting caused by limited sample size.

**Reviewer#4, Concern # 1 (The abstract should be presenting overview and key finding (both computational and biological) of the manuscript. Both current abstract and main text excessively focus on technical details on existing models and algorithm omitting the biological insights learnt from the new pipeline.):**

**Author response:** Thank you for your valuable comment. As a research team with a primary focus on computer science, our main contribution lies in developing novel computational models and algorithms for multi-omics data integration. While we recognize the importance of biological insights, our study aims to provide effective tools to improve feature representation and classification performance, which can subsequently support biological discoveries.

In the manuscript, we have demonstrated the biological relevance of our approach through improved classification accuracy across multiple cancer types, which suggests potential utility in understanding cancer heterogeneity. To better address your concern, we will enrich the abstract and discussion with concise statements highlighting the biological implications of our findings without compromising the technical focus of our work.

**Reviewer#4, Concern # 2 (The introduction states that "multi-omics models often prioritize stronger modalities at the expense of weaker ones.". Authors should explain more explicitly how proposed framework address this problem to achieved more balanced integration.):**

**Author response:** Thank you for your valuable feedback, First, we introduce a multi-head attention mechanism in the ViT encoder, which automatically allocates importance to different modality features based on their attention weights in the global context, avoiding reliance on dominant modalities. The representation of each modality is independently projected, normalized, and integrated, allowing the model to dynamically select useful features rather than statically favoring strong modalities. Additionally, we construct bidirectional generators (G1, G2) such that each modality is not only used to construct the latent space but is also forced to reconstruct information from another modality. This bidirectional adversarial structure, along with cycle consistency loss, ensures that even weak modalities with less information can have their latent representations optimized through the reconstruction mechanism, thus improving their effectiveness in the integration process. Finally, the supervised contrastive learning mechanism enhances modality synergy by pulling similar modality representations closer and pushing dissimilar ones apart, effectively reducing the risk of strong modalities monopolizing the representation space, and achieving a more balanced fusion.

In summary, our framework addresses the issue of "strong modality dominance and weak modality neglect" from multiple perspectives, including dynamic adjustment of modality weights through the attention mechanism, enhanced modality reconstruction via GANs, and optimized modality synergy through contrastive loss, thus achieving more balanced multi-omics data integration.

**Reviewer#4, Concern # 3 (The description of the CycleGAN architecture is not clear. For example the generator inputs are ambiguously defined. Authors need to revise.): Author response:** Thank you very much for your valuable feedback. We fully agree that a clear and detailed description of the architecture is essential for improving the logical flow and readability of the paper.

**Author action:** We have thoroughly revised the relevant section of the manuscript, specifically Section 2.2 CycleGAN Architecture, and have highlighted the changes in red. We have clearly defined the roles and inputs of each component in the CycleGAN framework.

We clarified that Generator G_1_ takes as input a latent representation z, which is extracted from the encoder $E$, and generates synthetic high-dimensional data $\tilde{x}$, i.e.,

G1: z → $\tilde{x}$

We also clarified that **Generator** G_2_ maps the original high-dimensional data x back to the latent space, producing $\tilde{z}$, i.e.,

G2:x→$\tilde{z}$

**Reviewer#4, Concern # 4 (The narratives of the manuscripts sometimes feel awkward and unprofessional. Authors should proofread the manuscript to make the logic flow.):**

**Author response:** Thank you for your detailed feedback. We highly value the language expression and logical structure of the manuscript. In response to your suggestions, we have carefully proofread and refined the entire text, with a focus on improving paragraph transitions and logical flow to enhance the overall professionalism and readability. We believe these improvements will make the paper clearer and more fluent, better conveying the research content and contributions.
